# Taxonomy and Broad-Spectrum Antifungal Activity of *Streptomyces* sp. SCA3-4 Isolated From Rhizosphere Soil of *Opuntia stricta*

**DOI:** 10.3389/fmicb.2019.01390

**Published:** 2019-06-28

**Authors:** Dengfeng Qi, Liangping Zou, Dengbo Zhou, Yufeng Chen, Zhufen Gao, Renjun Feng, Miaoyi Zhang, Kan Li, Jianghui Xie, Wei Wang

**Affiliations:** Institute of Tropical Bioscience and Biotechnology, Chinese Academy of Tropical Agricultural Sciences, Haikou, China

**Keywords:** *Streptomyces lilacinus*, Actinobacteria, banana *Fusarium* wilt, antifungal activity, biosynthetic genes, GC-MS

## Abstract

Actinobacteria are important producers of bioactive compounds. Extreme ecosystems cause evolution of novel secondary metabolic pathways of Actinobacteria and increase the possible discovery of new biological functions of bioactive compounds. Here, we isolated 65 Actinobacteria from rhizosphere soil samples of *Opuntia stricta*. An Actinobacteria strain (named SCA3-4) was screened against *Fusarium oxysporum* f. sp. *cubense* Tropical Race 4 (*Foc* TR4, ATCC 76255). The strain produced pink–white aerial mycelia and brown substrate mycelium on Gause No. 1 agar. Biverticillate chains of cylindrical spores were observed by scanning electron microscopy (SEM). Based on alignment of 16*S* rRNA sequences, a constructed phylogenetic tree showed that strain SCA3-4 shared a 99.54% similarity with *Streptomyces lilacinus* NRRL B-1968T. The morphological, biochemical, physiological, and molecular characteristics further indicated that strain SCA3-4 belongs to the *Streptomyces* sp. It can grow well on medium with the following antibiotics chloramphenicol, streptomycin, penicillin-G, gentamicin, erythromycin, nystatin or neomycin sulfate. The polymerase chain reaction (PCR) amplification of types I and II polyketide synthase genes (*PKS-I* and *PKS-II*) suggested its bioactive potential. Under treatment with 100 μg/ml of ethyl acetate extracts isolated from *Streptomyces* sp. SCA3-4, growth of *Foc* TR4 was inhibited and cell membrane was destroyed. Crude extracts also showed a broad-spectrum antifungal activity against 13 phytopathogenic fungi including *Foc* TR4 and displayed the lowest minimum inhibitory concentration (MIC) (0.781 μg/ml) against *Colletotrichum fragariae* (ATCC 58718). A total of 21 different compounds identified by gas chromatography–mass spectrometry (GC-MS) were composed of phenolic compound, pyrrolizidine, hydrocarbons, esters, and acids. Besides the known active compounds, *Streptomyces* sp. SCA3-4 possesses antimicrobial or other biological activities. Further attention will be paid on other compounds with no functional annotation, aiming at the discovery of new bioactive substances.

## Introduction

The secondary metabolites isolated from microbes exhibit either antimicrobial or antiviral activities, usually called “classical antibiotics.” Actually, the broadest definition of antibiotics should contain the bioactive compounds obtained from all living organisms (Bérdy, 2005). Antibiotics like penicillin and other antimicrobial agents have been widely used in agricultural, pharmaceutical, and industrial fields. However, overuse or misuse of antibiotics has given rise to the severe disease outbreaks caused by multiresistant pathogens in several countries (Sharma et al., 2016). Thus, it is urgent for researchers to seek novel and broad-spectrum antimicrobial metabolites from various sources including microbes (Hayakawa, 2008; Singh and Tripathi, 2011).

Actinobacteria are a large group of high G+C Gram-positive bacteria (Barka et al., 2016) and the primary sources of bioactive compounds in particular commercially available antibiotics (Barka et al., 2016; Rangseekaew and Pathom-aree, 2019). So far, more than 10,000 different bioactive compounds have been identified from Actinobacteria (Martins et al., 2019), for example, vancomycins from *Amycolatopsis orientalis*, rifamycin from *Amycolatopsis mediterranei*, teicoplanin from *Actinoplanes teichomyceticus*, erythromycin from *Saccharopolyspora erythraea*, and gentamicin from *Micromonospora purpurea* (Lancini and Lorenzetti, 1993; Lazzarini et al., 2000). In addition, several members of the Actinobacterial taxa were also identified to produce a wide range of chemical compounds, including alkaloid, polyene macrolide, saccharide, pyrazoloisoquinolinone, butenolide, nucleoside, etc. These metabolites demonstrated significant biological activities such as antifungal, antitumor, antibacterial, anti-inflammatory, and enzyme inhibition (Lim et al., 2003; Bérdy, 2005; Feng et al., 2007).

In natural soil habitat, *Streptomyces* are usually a major proportion of Actinobacteria (Tanaka and Omura, 1990; Kekuda et al., 2014). Nearly 45% of useful bioactive compounds currently known have been isolated from the genus *Streptomyces* (Azman et al., 2015). These secondary metabolites have potential roles for antimicrobials (Sacramento et al., 2004; Verma et al., 2009), antibacterials (Manikkam et al., 2014), antivirals (Sacramento et al., 2004), anticancer (Ser et al., 2017), antifungals (Oskay, 2009; Chen et al., 2018), herbicidals (Nakajima et al., 1991; Miller-Wideman et al., 1992), and antiparasitics (Pimentel-Elardo et al., 2010; Yao et al., 2014). Thus, novel secondary metabolites from the genus *Streptomyces* acting as drug candidates have been incomparable and seem to be almost inexhaustible (Bérdy, 2005). In the last decades, discovery efforts of multiple bioactive compounds have focused on *Streptomyces* from some terrestrial sources. However, novel compounds are difficult to be discovered from normal terrestrial *Streptomyces*, impelling to look for new metabolites from extreme environmental *Streptomyces* (Shivlata and Satyanarayana, 2015; Ser et al., 2016; Law et al., 2017; Lee et al., 2018). For this reason, the dry hot valley of Huili County (Sichuan Province, China) will be a potential region for the discovery of functional microbes due to a primitive ecosystem characterized by high temperature and low humidity. Therefore, isolation from extreme natural habitats is of interest to avoid re-isolation of *Streptomyces* that produce known bioactive metabolites.

Banana (*Musa* spp.) is one of the world’s most important fruits. Global banana industry is seriously threatened by *Fusarium* wilt (Wang et al., 2015). The disease is caused by the soil-borne fungi *Fusarium oxysporum* f. sp. *cubense* (*Foc*). Especially, a strain of *Foc* called Tropical Race 4 (TR4) has overcome more than 80% of global banana and plantain (Pérez-Vicente et al., 2014). Until now, there are few effective physical and chemical strategies to prevent the disease. Using microorganisms to control *Fusarium* wilt of banana is considered as a promising method (Wang et al., 2015). Our previous results showed that Actinobacteria are important microorganisms in the rhizosphere soil of banana and demonstrated the antagonism to phytopathogenic fungi including *Foc* TR4 (Chen et al., 2018). In the present study, we isolated an actinobacterium bioactive against *Foc* TR4 (ATCC 76255) from rhizosphere soil samples of *Opuntia stricta*. Compared by morphological, biochemical, physiological, and molecular characteristics, the strain was designated as *Streptomyces* sp. SCA3-4. We assessed its sensitivity to 10 antibiotics and broad-spectrum antifungal activity against 13 phytopathogenic fungi. Several biosynthesis genes of microbial natural products encoding polyketide synthases (*PKS-I* and *PKS-II*) and non-ribosomal peptide synthetases (NRPS) were amplified. Antifungal compounds were further analyzed by gas chromatography–mass spectrometry (GC-MS). Our aims were to discover microbial resources from the extreme ecosystem and evaluate their values in plant protection in the future agriculture.

## Materials and Methods

### Soil Sample Collection

The strain SCA3-4 was isolated from two soil samples (from 101°50′ 49″E to 26°3′ 18″ N and from 101°50′ 48″E to 26°3′ 17″) in a dry hot valley of the Huili County, Sichuan Province, China, in January 2014. The upper 20-cm rhizosphere soil samples of *O. stricta* were collected in sterile plastic bags and transported to the laboratory.

### Isolation of Actinobacteria

Actinobacteria were isolated using a serial dilution method (Williams and Davies, 1965) on starch casein agar (SCA) medium, including 10 g of soluble starch, 0.3 g of casein, 2.0 g of KNO_3_, 2.0 g of NaCl, 2.0 g of K_2_HPO_4_, 0.05 g of MgSO_4_ ⋅ 7H_2_O, 0.02 g of CaCO_3_, 0.01 g of FeSO_4_ ⋅ H_2_O, and 18 g of agar in 1 L of sterile water (pH 7.0–7.4). Potassium dichromate (50 mg/L) and nystatin (50 mg/L) were added to inhibit the growth of other bacteria and fungi, respectively. Briefly, soil samples were dried at room temperature and sieved with 0.425-mm mesh. One gram of soil sample and 9 ml of sterile water in a 10-ml tube were mixed and heated for 20 min in a waterbath at 55°C. Then, a serial dilution with 10^–2^, 10^–3^, and 10^–4^ was produced. One hundred microliters of each dilution was spread on the SCA medium and incubated at 28°C for 5–7 days. Representative colonies were selected and streaked on the YE agar medium (10 g of yeast extract, 10 g of malt extract, and 4 g of glucose in 1 L of distilled water, pH 7.3). These colonies were maintained on Gause No. 1 agar at 4°C and preserved in 20% glycerol broth at −80^∘^C for future use.

### Phytopathogenic Fungi

The antimicrobial activity was investigated against 13 target phytopathogenic fungi, including *Foc* TR4 (ATCC 76255), *F. oxysporum* f. sp. *cubense* Tropical Race 1 (*Foc* TR1; ACCC 31271), *F. oxysporum* f. sp. *cucumerinum* (ATCC 204378), *Colletotrichum fragariae* (ATCC 58718), *Colletotrichum musae* (ATCC 96726), *Colletotrichum gloeosporioides* (ATCC MYA-456), *Colletotrichum capsici* (ATCC 96158), *Curvularia fallax* (ATCC 34598), *Pyricularia oryzae* (ATCC 52352), *Fusarium graminearum* (ATCC MYA-4620), *Rhizoctonia solani* (ATCC 76144), *C. gloeosporioides* (ACCC 36351), and *Colletotrichum higginsianum* (ACCC 37053). These fungi were kindly provided by the Institute of Environment and Plant Protection, China Academy of Tropical Agricultural Sciences, Haikou, China.

### Screening Actinobacteria

Actinobacteria were screened according to the inhibition ability against *Foc* TR4 on potato dextrose agar (PDA) plates using the conventional spot inoculation method (Sadeghian et al., 2016; Chen et al., 2018). A mycelium plug (5-mm diameter) of target pathogens was placed in the center of PDA plates. Four mycelium blocks (5-mm diameter) of each actinobacterium were inoculated at four symmetrical points. The distance from the plate center was 26 mm. Three replicates were prepared for each actinobacterium. A plate with each target pathogen was used as the control. The colony diameters of the target pathogens were measured by the cross method after 5–7 days at 28°C (Yun et al., 2018). The inhibition zone and the percentage of fungal growth inhibition (GI) were calculated separately according to the following formula:

The⁢inhibition⁢zone=C-T

Percentage⁢growth⁢inhibition=[(C-T)/C]×100%

where *C* and *T* were the diameters of fungal mycelial growth in the control and treated plates, respectively (Aghighi et al., 2004). Finally, strain SCA3-4 was selected as a target strain according to its antifungal activity against *Foc* TR4.

### Morphological and Cultural Characteristics of Strain SCA3-4

Micromorphology of strain SCA3-4 was examined using the coverslip insertion method (Williams et al., 1989). The morphology of spore-bearing hyphae, spore surface, and spore chain was observed in a scanning electron microscope (ZEISS, Germany) as described by Kumar et al. (2014). Cultural characteristics of strain SCA3-4 were examined on PDA, Gause No. 1 agar, and various International Streptomyces Project (ISP) media, including yeast extract–malt extract agar (ISP_2_ or YE), oatmeal agar (ISP_3_), inorganic salts–starch agar (ISP_4_), glycerol–asparagine agar (ISP_5_), peptone yeast–iron agar (ISP_6_), and tyrosine agar (ISP_7_). Production of melanoid pigments was tested on the ISP_6_ and ISP_7_ media (Shirling and Gottlieb, 1966). The growth capability, pigment production, and color of both aerial and substrate mycelia were recorded after incubation at 28°C for 20 days. Colony colors were measured according to the Color Harmony Manual (Jacobson et al., 1958). To assign the appropriate genus to strain SCA3-4, we referred to *Bergey*’*s Manual of Systematic Bacteriology* (Williams et al., 1989)^1^.

### Physiological and Biochemical Characteristics of Strain SCA3-4

The growth conditions were assayed by changes of sole carbon and nitrogen sources, temperature (16–46°C), pH (4–10), and salt concentrations (1-9% NaCl, w/v) as described by Shirling and Gottlieb (1966). Carbon sources include D-xylose, D-mannitol, melezitose, L-rhamnose, L-arabinose, D-raffinose, maltose, sucrose, soluble starch, D-glucose, melibiose, D-ribose, inositol, salicin, D-cellobiose, D-galactose, trehalose, D-fructose, and xylan. Nitrogen sources include L-phenylalanine, ammonium sulfate, L-hydroxyproline, L(+)-cysteine, histidine, glycine, valine, ammonium oxalate, ammonium acetate, ammonium nitrate, ammonium molybdate, tetrahydrate, L-arginine, and glutamate. Indicators of hydrolysis of cellulose, starch, casein, Tween 20, Tween 80, gelatin liquefaction, nitrate reduction, and other biochemical tests were assessed according to the description of Sharma et al. (2016). Production of melanoid pigments was determined on the ISP6 and ISP7 media (Shirling and Gottlieb, 1966). Growth ability of strain SCA3-4 in the presence of 10 standard antibiotics was evaluated by the disc diffusion method (Williams et al., 1983; Kumar et al., 2014).

### Genomic DNA Extraction of Strain SCA3-4

Genomic DNA was extracted according to the method of Ahmad et al. (2017) with a slight modification. Briefly, strain SCA3-4 was cultivated in the YE liquid medium (pH 7.4) on a rotary shaker (150 rpm) at 28°C for 3 days. Culture solution (1.0 ml) was centrifuged at 9,000 rpm for 30 s. The pellets were resuspended in 480 μl of ethylenediaminetetraacetic acid (EDTA). Lysozyme (120 μl) was added and incubated at 37°C for 50 min. After centrifugation at 12,000 rpm for 2 min, the pellets were resuspended in 600 μl of lysis buffer and incubated at 80°C for 5 min. The cooled sample 1.8 μl of RNase A was added and incubated at 37°C for 15 min. Subsequently, 200 μl of phenol/chloroform (1:1) was then added and incubated on ice for 5 min. After centrifugation at 13,000 rpm at 4°C for 5 min, the supernatant (approximately 600 μl) was transferred into a clean 1.5-ml microcentrifuge tube and mixed with an equal volume of isopropanol. Seventy percent ethanol (0.5 ml) was added to the cleared supernatant and centrifuged at 12,000 rpm for 1 min. Finally, the pellets were air dried and resuspended in 100 μl of TE buffer (tris-hydrochloride buffer, pH 7.7, containing 1.0 mM EDTA). Genomic DNA was evaluated by 1% agarose gel electrophoresis and stored at −20^∘^C.

### PCR Amplification

The gene fragment of 16*S* rRNA from strain SCA3-4 was amplified using the pair of primers 27F (5′-AGAGTTTGATC CTGGCTCAG-3′) and 1492R (5′-GGTTACCTTGTTACGAC TT-3′) (Wang et al., 2015). A 50-ml reaction buffer contained 1 μl of 27F primer (10 mmol/L), 1 μl of 1492R primer (10 mmol/L), 25 μl of 2× Taq Master Mix, and 2 μl of genomic DNA. The PCR was performed in a TProfessional Trio PCR System (Biometra, Goettingen, Germany). The distilled water was used as a control template. The operation program included the initial denaturation at 94°C for 3 min followed by 31 cycles (94°C for 1 min, 56°C for 1 min, and 72°C for 2 min), and a final extension at 72°C for 10 min. The PCR product was analyzed in 1% (w/v) agarose gel electrophoresis and was sequenced by Beijing Liuhe Huada Gene Technology Co., Ltd. (Shenzhen, China).

### Phylogenetic Analysis

The similarity of the 16*S* rRNA sequence was compared with available sequences of bacteria obtained from the EzTaxon server database^2^ (Yoon et al., 2017) and the GenBank database. The multiple sequence alignment was carried out using CLUSTAL-W within BioEdit 7.0.5.3 (Thompson et al., 1994; Hall, 1999). A phylogenetic tree was constructed by the neighbor-joining method using MEGA 5.0 (Tamura et al., 2007). The confidence value of each clade was assessed using bootstrap analyses based on 1,000 replications (Felsenstein, 1985). The sequence was finally deposited in the GenBank database.

### Amplification of *PKS-I*, *PKS-II*, and *NRPS* Sequences

To obtain polyketide and peptide biosynthetic genes of *Streptomyces* sp. SCA3-4, the degenerate primers K1F (5′-TSAA GTCSAACATCGGBCA-3′) and M6R (5′-CGCAGGTTSCSG TACCAGTA-3′) were used to amplify the *PKS-I* gene with an expected product size of 1,200–1,400 bp (Ayuso-Sacido and Genilloud, 2005). The KS_α_ (5′-TSGCSTGCTTCGAYGCSATC-3′) and KS (5′-TGGAANCCGCCGAABCCGCT-3′) primers were used to amplify the *PKS-II* gene with an expected product size of 600 bp (Metsä-Ketelä et al., 1999). The A3F (5′-GCST ACSYSATSTACACSTCSGG-3′) and A7R (5′-SASGTCVCCSG TSCGGTAS-3′) primers were used to amplify the *NRPS* gene with an expected product size of 700 bp (Ayuso-Sacido and Genilloud, 2005). A total of 25 μl of reaction mixture contained 50 ng of template DNA, 12.5 μl of 2× PCR Master Mix, 0.5 μl of forward primer, 0.5 μl of reverse primer, and 10.5 μl of ddH_2_O. The amplification procedures included one denaturation step for 5 min at 95°C, followed by 35 cycles [94°C for 30 s, 55°C (for K1F-M6R and A3F-A7R) or 58°C (for KS_α_-KS) for 2 min, 72°C for 4 min], and a final extension at 72°C for 10 min (Passari et al., 2015).

### Extraction of Antifungal Compounds

To extract antifungal compounds, strain SCA3-4 was inoculated in a 5-L Erlenmeyer flask containing 1 L of fermentation broth (15 g of corn flour, 10 g of glucose, 0.5 g of K_2_HPO_4_, 0.5 g of NaCl, 0.5 g of MgSO_4_, 3 g of beef extract, 10 g of yeast extract, 10 g of soluble starch, 2 g of CaCO_3_, pH 7.2–7.4). The flask was cultured in a rotary shaker (150 rpm) at 28°C for 7 days. The fermentation broth was extracted with an equal volume of ethyl acetate. The mixture was filtered through a Whatman No. 1 filter and shaken vigorously in a separating funnel. Then, the collected organic solvent extract was evaporated using a rotary vacuum evaporator (EYELA, N-1300, Japan). The crude extract was dissolved in 10% dimethyl sulfoxide (DMSO) with a final concentration of 20.0 mg/ml. After filtering through a 0.22-μm sterile filter (Millipore, Bedford, MA, United States), the crude extract solution was kept in a refrigerator at 4°C for the antifungal bioassay and the GC-MS analysis.

### Antifungal Activity on the Mycelia Radial Growth

Crude extracts of *Streptomyces* sp. SCA3-4 were used to analyze the inhibition ability of the mycelial growth of 13 phytopathogenic fungi using the agar-well diffusion method as described by Tepe et al. (2005), Fontenelle et al. (2007) with a minor modification. The PDA solid medium was prepared in petri dishes (90-mm diameter). Four symmetrical wells with 26 mm from the center were punch by a sterile cork borer at the plate. One hundred microliters of crude extracts was delivered into each symmetrical well. Equivalent 10% DMSO was used as a control. A fungal block (5-mm diameter) was inoculated aseptically into the center of each petri dish. These plates were cultured at 28–30°C until the control mycelium covers the whole plate. The colony diameters of the pathogens were measured by the cross method (Yun et al., 2018). The inhibition zone and percentage of mycelial growth were calculated separately in terms of the following formula:

The⁢inhibition⁢zone=C-T

Growth⁢inhibition⁢percentage=[(C-T)/C]×100

where *C* is the average diameter of the pathogen colony in the control plate, and *T* is the average diameter of the tested pathogen colony in the treated plate (Pandey et al., 1982; Nimaichand et al., 2015). All experiments were performed in triplicates.

### Determination of Minimum Inhibitory Concentration of *Streptomyces* sp. SCA3-4

The minimum inhibitory concentrations (MICs) of crude extracts from *Streptomyces* sp. SCA3-4 against 13 phytopathogenic fungi were measured using a 96-well plate (Nunc MicroWell, untreated; Roskilde, Denmark) according to the description of Wang X.N. et al. (2013). The twofold serial dilutions of crude extracts were prepared for the MIC tests (50–0.391 μg/ml). Each well contained 80 μl of Roswell Park Memorial Institute (RPMI) mycological media, 100 μl of fungal suspension at 1.0 × 10^5^ CFU (colony-forming units)/ml, and 20 μl of crude extract solution. An equal volume of 10% DMSO was used as a negative control. Standard antibiotics such as cycloheximide and nystatin were served as positive controls. The 96-well plates were covered with a plastic lid and incubated at 28°C for 24 h. The absorbance of reaction solution was measured at 620 nm in a microplate photometer (Packard Spectra Count, Packard Instrument Co., Downers Grove, IL, United States). The lowest MICs with complete inhibition of growth were recorded.

### Effect of Crude Extracts on Spore Germination of *Foc* TR4

*Foc* TR4 was cultured on the PDA medium at 28°C for 7 days. Fungal spores were harvested by adding 5 ml of sterile water to each petri dish and rubbing the surface with a sterile L-shaped spreader (three times). The suspension was filtered through sterile muslin to remove mycelium. The spore concentration was determined using a Haemocytometer (Neubauer, Superior Ltd., Marienfield, Germany) and adjusted to a final concentration of 10^5^ CFU/ml. The extract solution (100 μg/ml) and the spore suspension were mixed at a ratio of 1:1 (v/v). The mixture (100 μl) was dripped in a cavity glass slide and incubated in a moist chamber at 28°C for 20 h. The experiment was repeated three times. A mixture of 10% DMSO and spore suspension was used as a control. One hundred spore germination of each slide was observed by an optical microscope (Axio Scope A1, Carl ZEISS, Germany). The percentage of spore germination (PSG) was calculated as the formula: PSG =(*A* - *B*)/*A* × 10, where *A* and *B* represent the spore germination rate of the control group and the treatment group, respectively (Chen et al., 2018).

### Effect of Crude Extracts on Cell Structure of *Foc* TR4

Potato dextrose agar medium containing 100 μg/ml of crude extract was prepared by pouring into sterilized petri dishes (90-mm diameter). An equal volume of 10% DMSO was used as a control. A disc (5-mm diameter) of *Foc* TR4 was inoculated aseptically into the center of each petri dish. These plates were cultured at 28°C for 7 days. The effect of crude extracts on *Foc* TR4 was detected by transmission electron microscopy (TEM) according to a previously published method (Lou et al., 2011). Briefly, the mycelium collected with a toothpick was added with 2.5% (v/v) glutaraldehyde in phosphate buffer solution (PBS, 0.1 mol/L, pH 7.3) and vacuumed until the sample sinks into the bottom of the bottle. The samples were fixed with fresh glutaraldehyde (2.5%, v/v) for 3 h and washed three times with PBS (0.1 mol/L, pH 7.0). Subsequently, samples were postfixed with 1% (w/v) osmium tetroxide in PBS (0.1 mol/L, pH 7.3) for 3 h at room temperature in a fume hood and were washed three times with PBS buffer. After gradual dehydration with the different concentrations of ethanol solutions (50, 70, 80, 90, 95, and 100% for 10 min, respectively), the samples were embedded in Epon 812 resin at 37°C for 12 h, 45°C for 12 h, and 60°C for 24 h, respectively. The embedded materials were sectioned with an ultramicrotome (EM UC6, Leica, Germany) at room temperature. Thereafter, the sections were double stained with saturated uranyl acetate and lead citrate and observed by TEM (HT7700, Hitachi, Japan) at an operating voltage of 80 kV.

### GC-MS Analysis of Crude Extracts

The GC-MS analysis of crude extracts was performed as previously described by Ser et al. (2015) and Supriady et al. (2015). Crude extracts were dissolved in spectroscopy-grade methanol and filtered through a 0.2-μm filter for GC-MS analysis. The equipment contains a Thermo Fisher Scientific trace (GC) equipped with a DSQ II (MS) and a DB-5MS capillary column (30.0 m × 0.25 mm × 0.25 μm). The sample was injected at 250°C using helium as carrier gas at 1 ml/min. The column temperature was programmed initially at 60°C for 1 min, followed by an increase of 5°C/min to 100°C. Then, it was kept isothermally for 5 min, ramped at 10°C/min to 250°C, held for 35 min, raised to 280°C at 8°C/min, and finally held for 25 min. The mass spectrometer was operated in the electron ionization mode at 70 eV with a continuous scan from 50 to 650 atomic mass units. The constituents were identified by matching the mass spectra with the National Institute of Standards and Technology (NIST, United States) library.

## Results

### Actinobacteria Isolation and Antifungal Activity Analysis Against *Foc* TR4

**FIGURE 1:**
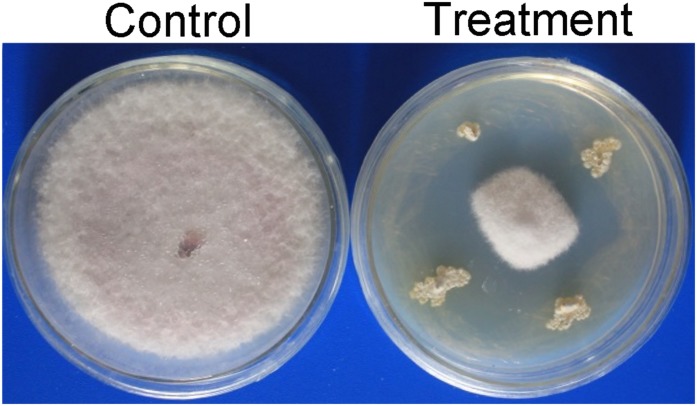
Inhibition of strain SCA3-4 on the mycelium growth of *Foc* TR4. Control, plate inoculated only with *Foc* TR4; Treatment, plate inoculated with *Foc* TR4 and strain SCA3-4.

A total of 65 Actinobacteria strains were isolated from two soil samples from the dry hot valley. All strains were screened for their antagonistic activity against *Foc* TR4 using a conventional spot inoculation method. Among them, 17 Actinobacteria showed antifungal activities against *Foc* TR4. Compared with the growth diameter (90.00 mm ± 0.00) of *Foc* TR4 in the control, the inhibition zone of strain SCA3-4 was 60.83 mm ± 1.26 (Figure 1). The percentage of mycelial inhibition was 67.59%. So, strain SCA3-4 was selected for further identification.

### Growth Characteristics and Morphology of Strain SCA3-4

**FIGURE 2:**
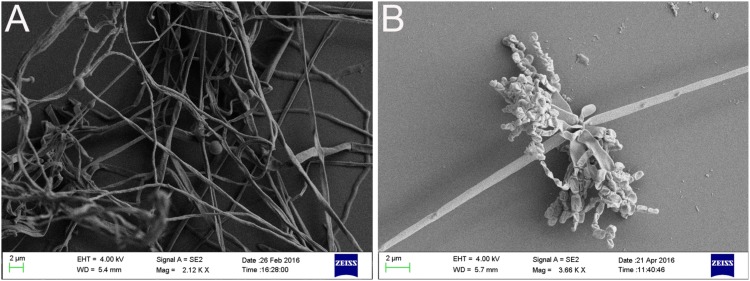
Morphologic characteristics of mycelium **(A)** as well as spore chain and spore **(B)** of strain SCA3-4.

After strain SCA3-4 was cultured on different media for 1 week at 28°C, we evaluated its vegetative and aerial mycelia as well as soluble pigments. The strain can grow well on eight culture media (Supplementary Figure 1) and produce soluble pigments on YE/ISP_2_, ISP_3_, ISP_6_, and PDA (Table 1). The SEM analysis revealed that strain SCA3-4 produced well-developed branch substrate and aerial mycelia. These biverticillate mycelia formed about six short branches at intervals. The top ends of the short branches produced about four spore chains with straight or hook shapes. Each chain was composed of 3 to 10 smooth and cylindrical spores (Figure 2).

**TABLE 1:** Cultural characteristics of strain SCA3-4.

**Medium**	**Aerial mycelium color**	**Substrate mycelium color**	**Soluble pigment**	**Growth**
Trytone-yeast extract agar (ISP_2_ or YE)	Pink white	Brown	Brown	+++
Oatmeal agar (ISP_3_)	Light–pink	Light–yellow	Brown	+++
Inorganic salts–starch agar (ISP_4_)	Pink white	Brown	None	+++
Glycerol–asparagine agar (ISP_5_)	Beige	Light–yellow	None	++
Peptone–yeast extract iron agar (ISP_6_)	Light–purple	Red–brown	Red	++
Tyrosine agar (ISP_7_)	Pink–gray	Brown	None	+++
Gause No. 1 agar	Pink–white	Brown	None	+++
PDA	Pink white	Brown	Brown	+++

*+++, good growth; ++, moderate growth.*

### Physiological and Biochemical Characteristics

Strain SCA3-4 belongs to aerobic Gram-positive bacteria. It could grow on the medium with pH 5.0–8.0 (optimum pH 7.0) at 21–44°C (optimum 28–43°C). Its tolerance to NaCl was less than 5% (w/v). Strain SCA3-4 could reduce nitrate, produce tyrosinase, liquefy gelatin, and degrade Tween 20 and 80. However, the strain was unable to produce urease, melanin, and H_2_S, nor hydrolyze starch and decompose cellulose (Table 2). It could fully utilize L-rhamnose, L-arabinose, D-raffinose, maltose, sucrose, soluble starch, D-glucose, melibiose, D-ribose, inositol, salicin, D-cellobiose, and xylan as sole source of carbon, respectively. Moreover, strain SCA3-4 could fully use L-phenylalanine, ammonium sulfate, L-hydroxyproline, L(+)-cysteine, histidine, glycine, valine, and ammonium oxalate as sole source of nitrogen, respectively. In addition, we also found that strain SCA3-4 showed resistance to seven antibiotics, including chloramphenicol, streptomycin, penicillin-G, gentamicin, erythromycin, nystatin, and neomycin sulfate, but showed sensitivity to ampicillin, tetracycline, and kanamycin sulfate (Table 3).

**TABLE 2:** Morphological, physiological, and biochemical characteristics of strain SCA3-4.

**Characteristics**	**Results**
*Morphological*	
Aerial mycelium color	Pink–white
Substrate mycelium color	Brown
Spore chain morphology	Biverticillate, straight, and short
Spore surface	Smooth
Spore shape	Cylindrical
*Physiological*	
Temperature range for growth (°C)	21–44 (optimum 28–43)
pH range for growth	5–8 (optimum 7)
NaCl tolerance for growth (%)	0–5
*Biochemical*	
Urease production	−
Tween 20	+
Tween 80	+
Degradation of cellulose	−
Melanoid pigment	−
Tyrosinase production	+
Starch hydrolysis	−
H_2_S production	−
Gelatin liquefaction	+
Nitrate reduction	+

**TABLE 3:** Nutrition Utilization and Antibiotic characteristics of strain SCA3-4.

**Characteristics**	**Results**
*Carbon source utilization*	
D-xylose	–
D-mannitol	–
L-arabinose	+
Soluble starch	+
Melezitose	–
D-glucose	+
Sucrose	+
L-Rhamnose	+
Maltose	+
D-raffinose	+
D-galactose	–
Melibiose	+
D-ribose	+
Inositol	+
Salicin	+
Trehalose	–
D-fructose	–
D-cellobiose	+
D-sorbitol	–
Xylan	+
*Nitrogen source utilization*	
L-phenylalanine	+
Ammonium sulfate	+
L-hydroxyproline	+
L(+)-cysteine	+
Histidine	+
Glycine	+
Valine	+
Ammonium oxalate	+
Ammonium acetate	–
Ammonium nitrate	–
Ammonium molybdate tetrahydrate	–
L-arginine	–
Glutamate	–
*Antibiotic sensitivity (μg/ml)*	
Ampicillin (0.5)	S
Chloramphenicol (1.5)	R
Streptomycin (0.5)	R
Penicillin-G (0.5)	R
Gentamicin (1.0)	R
Erythromycin (0.25)	R
Nystatin (5)	R
Tetracycline (1.5)	S
Neomycin sulfate (0.5)	R
Kanamycin sulfate (0.5)	S
Rifampicin (0.5)	S

*+, positive reaction; –, negative reaction; S, sensitivity; R, resistance.*

### Identification and Phylogenetic Analysis of Strain SCA3-4

**FIGURE 3:**
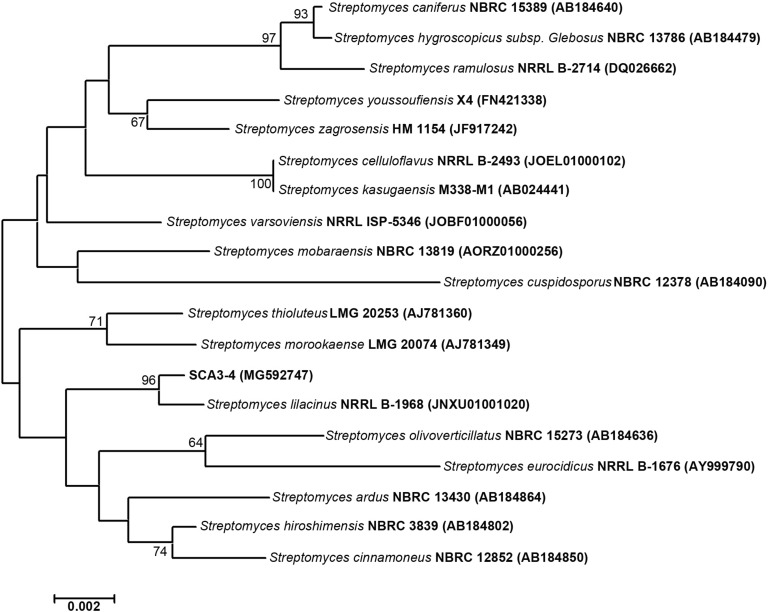
Construction of phylogenetic tree based on the 16*S* rRNA sequences from strain SCA3-4 and other selected strains. The phylogenetic tree was constructed using the neighbor-joining method. Numbers on the branches are bootstrap values calculated from 1,000 replicates. Bar, 0.002 substitutions per nucleotide position.

The 1,362 bp of the 16*S* rRNA partial sequence from strain SCA3-4 was amplified and sequenced. After alignment, the 16*S* rRNA sequence had the highest similarity with *Streptomyces lilacinus* NRRL B-1968T (99.54%). It was supported by the result of a phylogenetic tree constructed by the neighbor-joining method (Figure 3). The genomic data indicated that strain SCA3-4 belongs to the genus *Streptomyces* and was referred to as *Streptomyces* sp. SCA3-4. Subsequently, the 16*S* rRNA sequence of the strain was submitted to the GenBank database of NCBI with Accession Number MG592747.

### Amplification of the *PKS-I* and *PKS-II* Sequences

Many bioactive metabolites in Actinobacteria belong to polyketides, which are synthesized by polyketide synthases (*PKS-I* and *PKS-II*) in microbes (Novakova et al., 2004). Identification of the *PKS-I* and *PKS-II* genes is helpful to evaluate the potential of Actinobacteria to produce bioactive substances (Nimaichand et al., 2015). In our present study, *PKS-I* and *PKS-II* genes were amplified using two pairs of the K1F/M6R and KS_α_/KS primers, respectively. Sequencing results showed that a 1,249-bp sequence of *PKS-I* (Accession Number MK205361) and a 614-bp sequence of *PKS-II* (accession number MK205362) were obtained from *Streptomyces* sp. SCA3-4. By alignment, the *PKS-I* sequence showed the highest similarity (81%) with the homology gene (accession number WP 106676120.1) of the *Streptosporangium nondiastaticum* strain. The *PKS-II* sequence had a 99% similarity with the *PKS-II* gene from *Streptomyces cinnamoneus* subsp. *sparsus* strain (Accession Number BAF43372.1). The potential *NRPS* gene of *Streptomyces* sp. SCA3-4 could not be successfully amplified using the selected degenerate primers in our study.

### Antifungal Activity Assay of *Streptomyces* sp. SCA3-4

Phytopathogenic fungi can lead to critical diseases and serious yield losses in crops. To assess whether *Streptomyces* sp. SCA3-4 has a broad-spectrum antifungal activity, 13 phytopathogenic fungi were selected and screened against this strain. Our results demonstrated that crude extracts of *Streptomyces* sp. SCA3-4 could significantly inhibit mycelial growth of the 13 phytopathogenic fungi (Table 4 and Supplementary Figure 2). The antifungal activity expressed by the inhibition zones were shown as followed: *C. fragariae* (ATCC 58718) (84.67 ± 0.58), *C. gloeosporioides* (ACCC 36351) (63.33 ± 1.15), *P. oryzae* (ATCC 52352) (62.67 ± 2.08), *C. musae* (ATCC 96726) (54.00 ± 1.73), *F. oxysporum cucumerinum* (ATCC 204378) (50.00 ± 2.65), *C. higginsianum* (ACCC 37053) (46.33 ± 3.51), *Foc* TR4 (46.00 ± 1.00), *C. fallax* (ATCC 34598) (42.33 ± 1.53), *C. gloeosporioides* (ATCC MYA-456) (41.67 ± 2.89), *R. solani* (ATCC 76144) (41.67 ± 0.58), *C. capsici* (ATCC 96158) (40.33 ± 0.58), *Foc* TR1 (39.67 ± 0.58), and *F. graminearum* (ATCC MYA-4620) (36.67 ± 5.77). Maximum and minimum inhibition zones among the tested 13 pathogens were observed in *C. fragariae* (ATCC 58718) and *F. graminearum* Schwabe (ATCC MYA-4620), respectively. Therefore, crude extracts of *Streptomyces* sp. SCA3-4 exhibited a broad-spectrum antifungal activity.

**TABLE 4:** Inhibitory activities of *Streptomyces* sp. SCA3-4 against plant pathogenic fungi.

**Pathogenic fungi**	**Inhibition**	**Mycelial**
	**zone (mm)**	**inhibition (%)**
*Colletotrichum fragariae* (ATCC 58718)	$84.67\pm 0.58{}^{a}$	$94.07\pm 0.64{}^{a}$
*Colletotrichum gloeosporioides* (ACCC 36351)	$63.33\pm 1.15{}^{b}$	$70.37\pm 1.29{}^{b}$
*Pyricularia oryzae* (ATCC 52352)	$62.67\pm 2.08{}^{b}$	$69.63\pm 2.31{}^{b}$
*Colletotrichum musae* (ATCC 96726)	$54.00\pm 1.73{}^{c}$	$60.00\pm 1.92{}^{c}$
*Fusarium oxysporum* f. sp. *cucumerinum* (ATCC 204378)	$50.00\pm 2.65{}^{c,d}$	$55.56\pm 2.94{}^{c,d}$
*Colletotrichum higginsianum* (ACCC 37053)	$46.33\pm 3.51{}^{d,e}$	$51.48\pm 3.90{}^{d,e}$
*Fusarium oxysporum* f. sp. *cubense* Tropical Race 4 (ATCC 76255)	$46.00\pm 1.00{}^{d,e}$	$51.11\pm 1.11{}^{d,e}$
*Curvularia fallax* (ATCC 34598)	$42.33\pm 1.53{}^{e,f}$	$47.04\pm 1.70{}^{e,f}$
*Colletotrichum gloeosporioides* (ATCC MYA-456)	$41.67\pm 2.89{}^{f}$	$46.29\pm 3.21{}^{f}$
*Rhizoctonia solani* (ATCC 76144)	$41.67\pm 0.58{}^{f}$	$46.30\pm 0.64{}^{f}$
*Colletotrichum capsici* (ATCC 96158)	$40.33\pm 0.58{}^{f,g}$	$44.81\pm 0.65{}^{f,g}$
*Fusarium oxysporum* f. sp. *cubense* Tropical Race 1 (ATCC 76244)	$39.67\pm 0.58{}^{f,g}$	$44.07\pm 0.64{}^{f,g}$
*Fusarium graminearum* (ATCC MYA-4620)	$36.67\pm 5.77{}^{g}$	$40.74\pm 6.41{}^{g}$

*Data in the table are means ± SD. Different letters in the same column represent significant difference at *p* < 0.05 by Duncan’s test.*

### MIC of *Streptomyces* sp. SCA3-4

Minimum inhibitory concentration values of crude extracts from *Streptomyces* sp. SCA3-4 against 13 phytopathogenic fungi were determined using a 96-well microtiter assay. The lowest MIC was 0.781 μg/ml against C. *fragariae* (ATCC 58718), suggesting that crude extracts had a strong inhibitory activity against this strain. In addition, we also found that crude extracts also exhibit better antifungal activity against *F. oxysporum cucumerinum* (ATCC 204378), *F. oxysporum* Tropical Race 4 (ATCC 76255), *C. capsici* (ATCC 96158), and *F. oxysporum* Tropical Race 1 (ATCC 76244) with 1.563 μg/ml of MIC. The highest MIC (25 μg/ml) was observed against *P. oryzae* (ATCC 52352) and *R. solani* (ATCC 76144). Treatment of 10% DMSO in control had no inhibitory efficiency for the phytopathogenic fungi (Table 5).

**TABLE 5:** Minimum inhibitory concentration values of crude extracts of *Streptomyces* sp. SCA3-4 against 13 pathogenic fungi.

**Pathogenic fungi**	**MIC of SCA3–4 (μg/ml)**	**MIC of *Cy* (μg/ml)**	**MIC of *Ny* (μg/ml)**
*Colletotrichum fragariae* (ATCC 58718)	>0.781	>12.5	>25
*Colletotrichum gloeosporioides* (ACCC 36351)	>6.25	>0.391	>25
*Pyricularia oryzae* (ATCC 52352)	>25	>50	>25
*Colletotrichum musae* (ATCC 96726)	>12.5	> 0.391	>25
*Fusarium. oxysporum* f. sp. *cucumerinum* (ATCC 204378)	>1.563	>25	>12.5
*Colletotrichum Higginsianum* (ACCC 37053)	>12.5	>0.781	>50
*Fusarium oxysporum* f. sp. *cubense* Tropical Race 4 (ATCC 76255)	>1.563	>1.563	>6.25
*Curvularia fallax* (ATCC 34598)	>12.5	>3.125	>12.5
*Colletotrichum gloeosporioides* (ATCC MYA-456)	>3.125	>12.5	>25
*Rhizoctonia solani* (ATCC 76144)	>25	>12.5	>0.391
*Colletotrichum capsici* (ATCC 96158)	>1.563	>50	>25
*Fusarium oxysporum* f. sp. *cubense* Tropical Race 1 (ATCC 76244)	>1.563	>6.25	>12.5
*Fusarium graminearum* (ATCC MYA-4620)	>6.25	>25	>25

**Cy*, *Cycloheximide* (antifungal agent); *Ny*, *Nystatin* (antifungal agent).*

### Inhibition Efficiency of Crude Extracts for Spore Germination of *Foc* TR4

**FIGURE 4:**
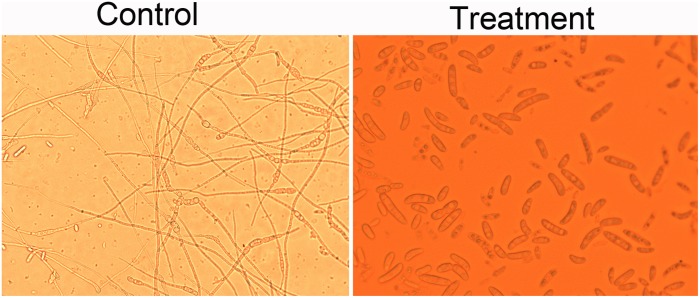
Effects of the crude extracts of *Streptomyces* sp. SCA3-4 on spore germination of *Foc* TR4. Control, treatment with 10% DMSO; Treatment, treatment with the crude extracts (100 μg/ml) of *Streptomyces* sp. SCA3-4.

Effect of crude extracts of *Streptomyces* sp. SCA3-4 on spore germination of *Foc* TR4 was shown in Figure 4. The germination rate in the control and treatment groups was 90.00 ± 2.00% and 5.33 ± 0.58%, respectively. The inhibition PSG against *Foc* TR4 was 94.08%.

### Effect of Crude Extracts on the Cell Structure of *Foc* TR4

**FIGURE 5:**
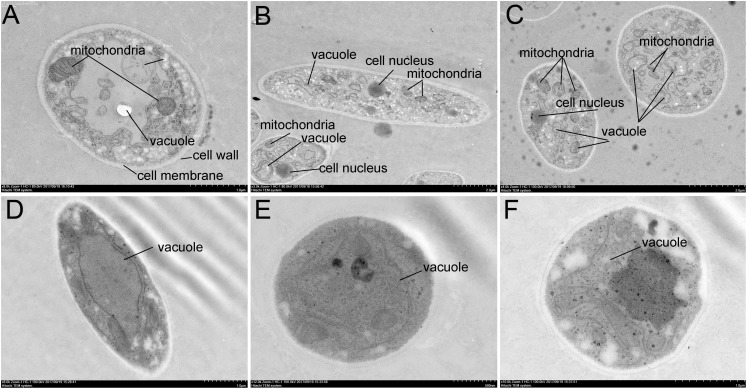
Transmission electron microscopy micrographs of *Foc* TR4 treated with crude extracts (100 μg/ml) of *Streptomyces* sp. SCA3-4. **(A–C)** Treatment with 10% DMSO. **(D–F)** Treatment with the crude extracts.

To further confirm antibacterial activity of *Streptomyces* sp. SCA3-4, morphology changes of *Foc* TR4 were detected by TEM (Figure 5). In the control group, the cell wall and membrane were intact and well defined. The organelles such as mitochondria and endoplasmic reticulum were structurally well defined (Figures 5A–C). On the contrary, cells treated with crude extracts of *Streptomyces* sp. SCA3-4 exhibited that number of organelles in the cytoplasm were disintegrated, and cell membrane was dissolved (Figures 5D–F).

### GC-MS Analysis of Crude Extracts

**FIGURE 6:**
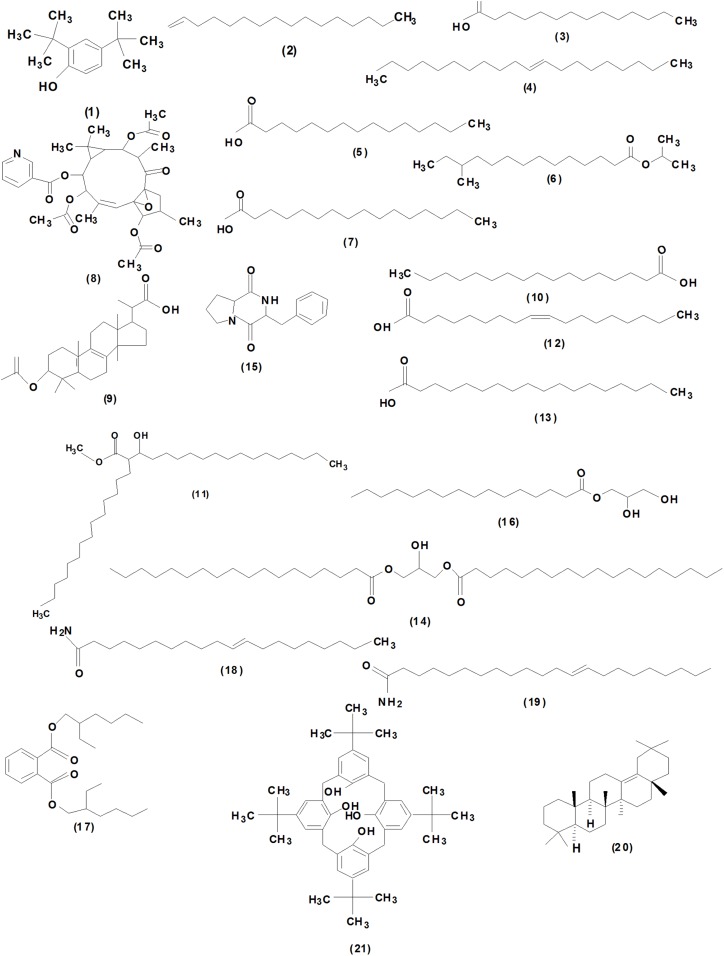
Chemical structures of the identified compounds from crude extracts of *Streptomyces* sp. SCA3-4.

The chemical composition of the SCA3-4 crude extract was analyzed by GC-MS (Supplementary Figure 3). Twenty-one chemical compounds were identified by the comparison of their mass spectra against the NIST library based on retention time, molecular mass, and molecular formula (Supplementary Table 1). Their chemical structures are shown in Figure 6. By contrast, these compounds were identified as 2,4-bis (1,1-dimethylethyl)-phenol (1), 1-hexadecene (2), tetradecanoic acid (3), (*E*)-9-eicosene (4), pentadecanoic acid (5), i-propyl 12-methyltetradecanoate (6), *N*-hexadecanoic acid (7), (10*Z*)-4,9,13-triacetoxy-3,6,6,10,14-pentamethyl-2-oxo-16-oxatetracyclo [10.3.1.01,12.05,7] hexadec-10-en-8-yl nicotinate (8), 2-(3-acetoxy-4,4,14-trimethylandrost-8-en-17-yl)-propanoic acid (9), heptadecanoic acid (10), 3-hydroxy-2-tetradecyl-octadecanoic acid methyl ester (11), oleic acid (12), octadecanoic acid (13), octadecanoic acid, 2-hydroxy-1,3-propanediyl ester (14), hexahydro-3-(phenylmethyl) pyrrolo[1,2-a]pyrazine-1,4-dione (15), hexadecanoic acid, 2,3-dihydroxypropyl ester (16), bis(2-ethylhexyl) phthalate (17), *trans*-11-eicosenamide (18), (*Z*)-13-docosenamide(19), olean-13 (18) ene (20), and pentacyclo[19.3.1.1(3,7).1(9,13).1(15,19)]octacosa-1(25),3,5,7(28), 9,11,13(27),15,17,19(26),21,23-dodecaene-25,26,27,28-tetrol, 5, 11,17,23-tetrakis (1,1-dimethylethyl) (21). The peak areas of compounds represented the quantity proportions in the crude extracts of *Streptomyces* sp. SCA3-4 (Supplementary Figure 3).

## Discussion

Due to overuse of antibiotics, environmental pollution, and resistance increase of bacteria and fungi against these compounds, we require the discovery of new biological functions of bioactive compounds. Actinobacteria are important producers of antibiotics and other important bioactive substances. Accumulated data indicated that the extreme ecosystems may cause evolution of novel secondary metabolic pathways of Actinobacteria and increase the discovery possibility of new microbial products (Cai et al., 2009; Bull, 2010; Shivlata and Satyanarayana, 2015). In our study, we isolated an actinobacterium SCA3-4 strain from the rhizosphere soil of *O. stricta* collected from the dry hot valley of Huili County in Sichuan Province, China. There is an extreme environment with high temperature and low humidity and a less-explored ecosystem. Based on the 16*S* rRNA sequence analysis as well as the morphological, cultural, physiological, and biochemical characteristics, strain SCA3-4 was identified as belonging to the genus *Streptomyces*. *Streptomyces* species have attracted extensive attention due to the tremendous success of their natural products in practical application. The genus *Streptomyces* has been studied widely in the past decades and was previously reported for the production of antimicrobial and anticancer compounds (Suzuki et al., 1982; Li et al., 2006; Wang C.L. et al., 2013). By alignment of the sequenced 16*S* rRNA, the *Streptomyces* sp. SCA3-4 has the highest similarity with *S. lilacinus* NRRL B-1968T. Until now, there are few studies related to the antimicrobial activity of *S. lilacinus* NRRL B-1968T strain (Nakazawa et al., 1956). We found that *Streptomyces* sp. SCA3-4 could be a potential strain for the development of antifungal drugs against a wide range of pathogenic fungi including *Foc* TR4 (Table 4 and Supplementary Figure 2).

Accumulated evidence indicates that synthesis of bioactive compounds is closely related with the *PKS* and *NRPS* genes (Ginolhac et al., 2004; Albright et al., 2014). The PKS pathway is responsible for the biosynthesis of fatty acids in some bacteria (Takeyama et al., 1997; Donadio et al., 2007). A PCR-based amplification of biosynthetic genes is a powerful tool for the prediction of Actinobacteria with the potential ability of producing bioactive secondary metabolites (Hornung et al., 2007). In our study, two *PKS* genes of *Streptomyces* sp. SCA3-4 were amplified using the degenerate primers and classified into type *PKS-I* and type *PKS-II*, respectively (Novakova et al., 2004). The type *PKS-I* genes encode multifunctional proteins with several active domains. They are responsible for the assembly and modification of the polyketide carbon chain as well as the formation of complex macrolide polyketides (Ginolhac et al., 2004). The type *PKS-II* genes encode several monofunctional proteins with a similar function in the synthesis of cyclic aromatic polyketides (Hopwood, 1997). Similarly, the two PKS types were also identified in other Actinobacteria (Metsä-Ketelä et al., 1999; Qin et al., 2009; Albright et al., 2014). However, we did not amplify the *NRPS* gene. This is probably because the degenerate primers might not be suitable for amplification of the *NRPS* gene. The other reason is the absence of NRPS in *Streptomyces* sp. SCA3-4. It was supported by that the *NRPS* genes could not be detected in 34 strains of tested 46 Actinobacteria in a previous study (Qin et al., 2009).

To further identify the antifungal products of *Streptomyces* sp. SCA3-4, the GC-MS method was used to analyze the crude extracts. Twenty-one chemical compounds were detected, including phenol, pyrrolizidine, hydrocarbons, esters, and acids (Figure 6 and Supplementary Table 1). Tetradecanoic acid (myristic acid), pentadecanoic acid, *N*-hexadecanoic acid (palmitic acid), heptadecanoic acid, oleic acid, and octadecanoic acid (stearic acid) contain the typical characteristics of fatty acids with a carboxyl group (-COOH) and a methyl group (-CH3) in the two ends of an aliphatic hydrocarbon chain (Desbois and Smith, 2010). Actually, fatty acids have been identified as active ingredients of the antimalarial, antimycobacterial, and antifungal properties in ethnic and herbal medicines (Carballeira, 2008; Desbois and Smith, 2010; Pohl et al., 2011). In addition, tetradecanoic acid (myristic acid), *N*-hexadecanoic acid (palmitic acid), and octadecanoic acid (stearic acid) also possess antimicrobial activity against different plant pathogens (Altieri et al., 2007; Liu et al., 2008; Liu and Huang, 2012; Johannes et al., 2016). Furthermore, *N*-hexadecanoic acid also functions as an anti-inflammatory agent (Aparna et al., 2012). Oleic acid significantly inhibits the mycelial growth of *Pyrenophora ultimum* and *Crinipellis perniciosa* (Walters et al., 2004). It is worth mentioning that (*Z*)-13-docosenamide occupies 44.34% of the total constituents in *Streptomyces* sp. SCA3-4. The metabolite exhibited significant antifungal and antitumor properties in the ether extracts of endophytic fungus *Paecilomyces* sp. (Donio et al., 2013). The (*Z*)-13-docosenamide also participates in the regulation of the central nervous system in antagonizing depression and anxiety (Li et al., 2017). Therefore, we speculated that these chemical compounds isolated from *Streptomyces* sp. SCA3-4 may be involved in antifungal activity. It was further evidenced by cell structure changes of *Foc* TR4 treated with crude extracts of the strain (Figures 5D–F). The antifungal mechanism of chemical compounds may destroy cell structure and inhibit the germination and growth of fungal spores (Makarieva et al., 2002). Interestingly, the different length and saturability of fatty acids were also detected in the GC-MS fractions of *Streptomyces* sp. SCA3-4. It is revealed that saturated fatty acids with 10 to 12 carbon atoms have the strongest antimicrobial activity (Desbois and Smith, 2010). The antimicrobial ability of saturated fatty acids with a longer or shorter carbon chain tends to be decreased (Sun et al., 2003; Wille and Kydonieus, 2003). Under the same length of carbon chain, unsaturated fatty acids possess greater potency of antimicrobes than saturated fatty acids (Zheng et al., 2005; Desbois et al., 2008). However, whether they are related to the antimicrobial ability is still an open question.

In addition, 1-hexadecene,2-(3-acetoxy-4,4,14-trimethylandrost-8-en-17-yl)-propanoic acid, and 2,4-bis(1,1- dimethylethy)-phenol were also detected in the crude extracts of *Streptomyces* sp. SCA3-4. 1-Hexadecene has been reported to possess antibacterial activity (Beevi et al., 2014). 2-(3-Acetoxy-4, 4,14-trimethylandrost-8-en-17-yl)-propanoic acid was identified as an inhibition factor for the activity of protein tyrosine phosphatase 1B, which is a negative regulator of the insulin-signaling pathway (Venkatachalam et al., 2013). It was also found that the compound possesses antimicrobial and antitumor activities (Venkatachalam et al., 2013). 2,4-Bis(1,1-dimethylethy)-phenol had antifungal (Rangel-Sánchez et al., 2014) and anticancer activities (Rajaram et al., 2013). A high concentration of pyrrolo[1,2-a] pyrazine-1,4-dione was also observed in the crude extracts. The previous studies indicated that it had strong antioxidant and antimicrobial activities against *Escherichia coli*, *Pseudomonas aeruginosa*, *P. oryzae*, and *Enterococcus faecalis* (Melo et al., 2014; Awla et al., 2016). It is worth noting that compounds with no functional annotation in the GC-MS fractions may also contribute to antifungal activity and need to been elucidated in further studies.

## Conclusion

In this study, strain SCA3-4 was isolated from the rhizosphere soil of *O. stricta* in a dry hot valley by the serial dilution technique. Based on the homology alignment of 16*S* rRNA and the morphological, cultural, physiological, and biochemical characteristics, the strain was identified as *Streptomyces* sp. The *Streptomyces* sp. SCA3-4 exhibited a strong antagonistic ability against *Foc* TR4 and resistance to seven antibiotics including chloramphenicol, streptomycin, penicillin-G, gentamicin, erythromycin, nystatin, and neomycin sulfate. Two genes, *PKS-I* and *PKS-II*, related to the synthesis of the antifungal compounds were amplified. Ethyl acetate extracts exhibited a broad-spectrum antifungal activity against 13 plant pathogenic fungi and displayed the lowest MIC (0.781 μg/ml) against *C. fragariae* (ATCC 58718) and the highest MIC (25 μg/ml) against *P. oryzae* (ATCC 52352) and *R. solani* (ATCC 76144). Especially, treatment of crude extracts inhibited spore germination, destroyed structures of cell membrane, and decreased the number of organelles in the cytoplasm of *Foc* TR4. The GC-MS analysis revealed that 21 different compounds were identified from *Streptomyces* sp. SCA3-4. These compounds composed of phenolic compound, pyrrolizidine, hydrocarbons, esters and acids (Figure 6 and Supplementary Table 1) might contribute to antimicrobial or other biological activities.

## Data Availability

No datasets were generated or analyzed for this study.

## Author Contributions

DQ designed the study and carried out the experiments. JX and WW supervised the research work and guided the experimental design. LZ and DZ provided suggestions for the research work. LZ, YC, RF, MZ, ZG, and KL were involved in some experiments such as soil sampling, isolation, and identification of strains. DQ and YC analyzed the data. DQ and WW prepared the final version of the manuscript.

## Conflict of Interest Statement

The authors declare that the research was conducted in the absence of any commercial or financial relationships that could be construed as a potential conflict of interest.

## Footnotes

1 http://www.bacterio.net/

2 https://www.ezbiocloud.net/identify, data released in March 2019
