# Supplementary material for: Taxonomy and Broad-Spectrum Antifungal Activity of Streptomyces sp. SCA3-4 Isolated From Rhizosphere Soil of Opuntia stricta
Source: Front Microbiol. 2019 Jun 28;10:1390. doi: 10.3389/fmicb.2019.01390 (PMC6609889; doi:10.3389/fmicb.2019.01390)
Supplement: Supplementary file 1 [file Table_1.DOC]

Supplementary Material

## Supplementary Figure


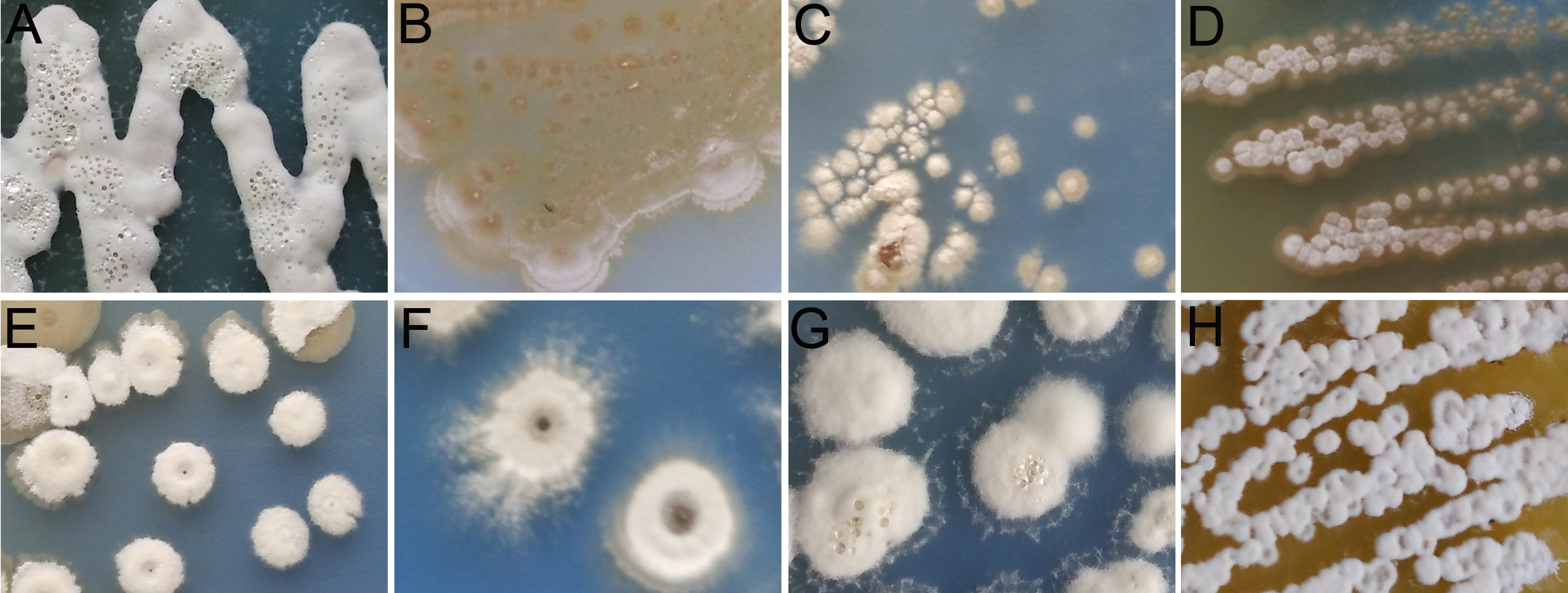


**Supplementary Figure 1.** Growth characteristics of strain SCA3-4 on eight media.

A: YE, B: ISP3, C: ISP5, D: ISP6, E: ISP4, F: ISP7, G: Gause No.1 agar, H: PDA.

**
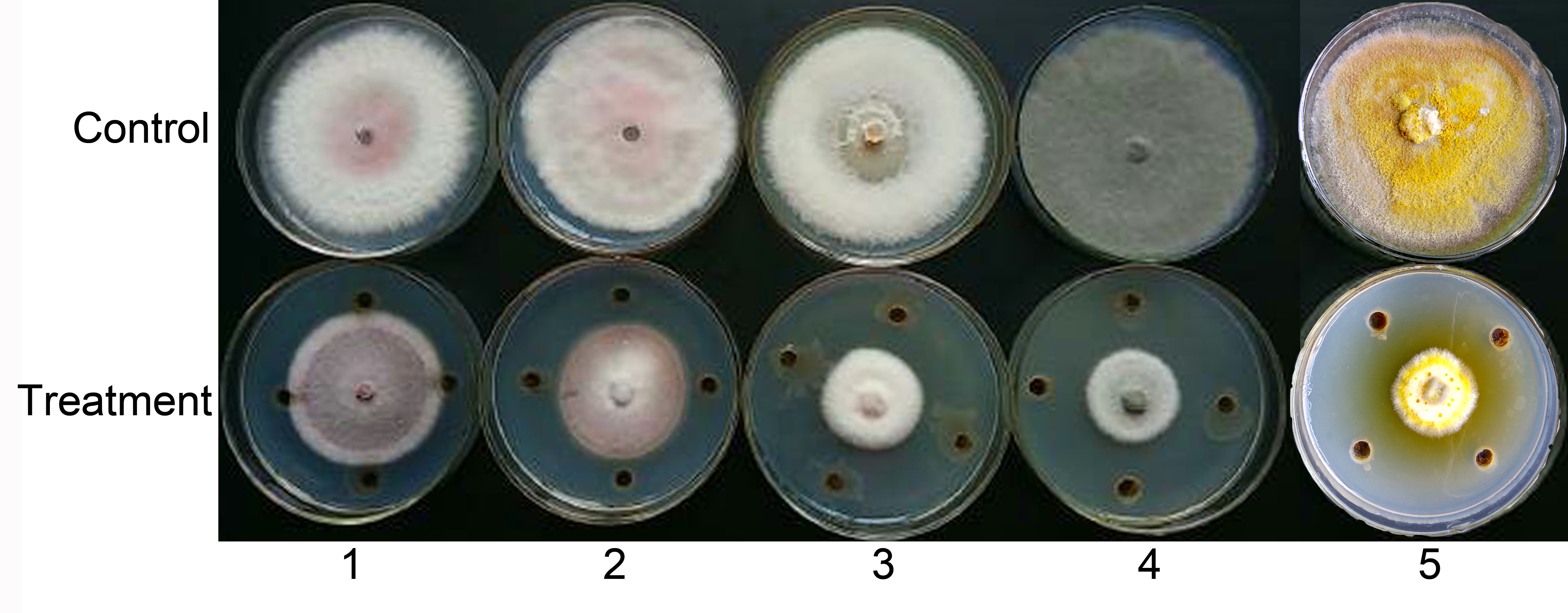
**

**Supplementary Figure 2.** Inhibition effect of crude extracts of *Streptomyces* sp. SCA3-4 on mycelium of plant pathogenic fungi.

1: *Colletotrichum gloeosporioides* (ATCC MYA-456), 2: *Foc* TR4 (ATCC 76255), 3: *Fusarium oxysporum* f. sp. *Cucumerinum* (ATCC 204378), 4: *Colletotrichum gloeosporioides* (ACCC 36351), 5: *Pyricularia oryzae* (ATCC 52352).

**Supplementary Figure 3.** Chemical composition analysis of crude extracts isolated from *Streptomyces* sp. SCA3-4 by GC-MS

**Supplementary Table 1 Compounds identified from crude extracts of *Streptomyces* sp. SCA3-4 by GC-MS.**

| **Compounds** | **Probability (%)** | **RT (min)** | **MW** | **Area (%)** | **MF** | **Activity** | **References** |
| --- | --- | --- | --- | --- | --- | --- | --- |
| 2,4-bis(1,1-dimethylethy)-phenol | 52.76 | 21.63 | 206 | 1.57 | C_14_H_2_2O | antifungal, anticancer | Rangel-Sánchez et al., 2014; Rajaram et al., 2013 |
| 1-hexadecene | 5.22 | 22.69 | 224 | 0.64 | C_16_H_32_ | antibacterial | Beevi et al., 2014 |
| Tetradecanoic acid | 23.88 | 24.38 | 228 | 0.37 | C_14_H_28_O_2_ | antibacterial, antifungal, | Liu et al., 2012; Liu et al.,2008; Altieri et al., 2007; Kabara et al., 1972 |
| (E)-9-eicosene | 3.36 | 25.1 | 280 | 0.63 | C_20_H_40_ | no activity reported |  |
| Pentadecanoic acid | 64.2 | 25.51 | 242 | 1.15 | C_15_H_30_O_2_ | no activity reported |  |
| i-propyl 12-methyl tetradecanoate | 42.99 | 25.61 | 284 | 1.36 | C_18_H_36_O_2_ | no activity reported |  |
| *N*-Hexadecanoic acid | 78.75 | 27.01 | 256 | 24.70 | C16H32O2 | antibacterial, antifungal,  anti-inflammatory | Liu et al.，2012; Liu et al.,2008; Altieri et al., 2007; Aparna et al.，2012 |
| (10Z)-4,9,13-Triacetoxy-3,6,6,10,14-pentamethyl-2-oxo-16-oxatetracyclo[10.3.1.01,12.05,7]hexadec-10-en-8-yl nicotinate | 8.00 | 27.2 | 597 | 1.61 | C_32_H_39_NO_10_ | no activity reported |  |
| 2-(3-acetoxy-4,4,14-trimethylandrost-8-en-17-yl)-propanoic acid | 26.8 | 27.38 | 430 | 0.32 | C_27_H_42_O_4_ | PTP 1B inhibitory action, anti-microbial and anti-tumor | Venkatachalam et al., 2013 |
| Heptadecanoic acid | 79.05 | 27.58 | 270 | 3.05 | C_17_H_34_O_2_ | no activity reported |  |
| 3-hydroxy-2-tetradecyl-octadecanoic acid methyl ester | 19.64 | 27.93 | 510 | 0.59 | C_33_H_66_O_3_ | no activity reported |  |
| Oleic Acid | 16.36 | 28.68 | 282 | 5.65 | C_18_H_34_O_2_ | antifungal | Walters et al., 2004 |
| Octadecanoic acid | 28.92 | 28.89 | 284 | 4.7 | C_18_H_36_O_2_ | antibacterial | Liu et al., 2012 |
| Octadecanoic acid, 2-hydroxy-1,3-propanediyl ester | 16.84 | 29.07 | 624 | 0.89 | C_39_H_76_O_5_ | no activity reported |  |
| Hexahydro-3- (phenylmethyl) pyrrolo[1,2-a]pyrazine-1,4- dione | 60.5 | 31.98 | 244 | 0.33 | C_14_H_16_N_2_O_2_ | antibacterial | Melo et al., 2014; Awla et al., 2016 |
| Hexadecanoic acid, 2,3-dihydroxypropyl ester | 31.09 | 33.37 | 330 | 0.44 | C_19_H_38_O_4_ | no activity reported |  |
| bis(2-ethylhexyl) phthalate | 7.99 | 33.63 | 390 | 0.27 | C_24_H_38_O_4_ | no activity reported |  |
| Trans-11-eicosenamide | 15.34 | 34.5 | 309 | 0.57 | C_20_H_39_NO | no activity reported |  |
| (Z)-13-Docosenamide | 90.72 | 40.36 | 337 | 37.08 | C_22_H_43_NO | antiviral,Antidepressant | Donio et al., 2013; Li et al., 2017 |
| Olean-13(18)-ene | 10.8 | 68.64 | 410 | 0.86 | C_30_H_50_ | no activity reported |  |
| Pentacyclo[19.3.1.1(3,7). 1(9,13).1(15,19)]octacosa-1(25),3,5,7(28),9,11,13(27),15,17,19(26),21,23-dodecaene-25,26,27,28-tetrol, 5,11,17,23-tetrakis(1 1 dimethylethyl) | 58.82 | 82.21 | 648 | 1.06 | C_44_H_56_O_4_ | no activity reported |  |

RT is retention time; MW is molecular weight of compounds; MF is Molecular Formula.
